# Profiling inflammatory markers in patients with pneumonia on intensive care

**DOI:** 10.1038/s41598-018-32938-6

**Published:** 2018-10-03

**Authors:** David B. Antcliffe, Arnaud M. Wolfer, Kieran P. O’Dea, Masao Takata, Elaine Holmes, Anthony C. Gordon

**Affiliations:** 1 Section of Anaesthetics, Pain Medicine and Intensive Care, Imperial College London, London, UK; 2 Section of Computational and Systems Medicine, Department of Surgery and Cancer, Faculty of Medicine, Imperial College London, London, UK

## Abstract

Clinical investigations lack predictive value when diagnosing pneumonia, especially when patients are ventilated and develop ventilator associated pneumonia (VAP). New tools to aid diagnosis are important to improve outcomes. This pilot study examines the potential for a panel of inflammatory mediators to aid in the diagnosis. Forty-four ventilated patients, 17 with pneumonia and 27 with brain injuries, eight of whom developed VAP, were recruited. 51 inflammatory mediators, including cytokines and oxylipins, were measured in patients’ serum using flow cytometry and mass spectrometry. The mediators could separate patients admitted to ICU with pneumonia compared to brain injury with an area under the receiver operating characteristic curve (AUROC) 0.75 (0.61–0.90). Changes in inflammatory mediators were similar in both groups over the course of ICU stay with 5,6-dihydroxyeicosatrienoic and 8,9-dihydroxyeicosatrienoic acids increasing over time and interleukin-6 decreasing. However, brain injured patients who developed VAP maintained inflammatory profiles similar to those at admission. A multivariate model containing 5,6-dihydroxyeicosatrienoic acid, 8,9-dihydroxyeicosatrienoic acid, intercellular adhesion molecule-1, interleukin-6, and interleukin-8, could differentiate patients with VAP from brain injured patients without infection (AUROC 0.94 (0.80–1.00)). The use of a selected group of markers showed promise to aid the diagnosis of VAP especially when combined with clinical data.

## Introduction

Pneumonia is a common cause for admission to the Intensive Care Unit (ICU) and ventilator associated pneumonia (VAP) is a common complication in patients requiring mechanical ventilation, occurring in 8–28% of such patients^1,2^. Development of VAP is associated with increased mortality, morbidity, longer intensive care unit and hospital stays, and increased healthcare costs^2–4^.

VAP diagnosis remains challenging. Clinical^5,6^, radiological^6,7^ and laboratory findings^6,8^ lack sensitivity and specificity. Biomarkers such as C-reactive protein (CRP)^9^, procalcitonin^10^, and soluble triggering receptor expressed on myeloid cells (sTREM-1)^11,12^ have either failed to show strong diagnostic benefit or have failed to be widely adopted. The inability to accurately diagnose pneumonia leads to increased morbidity with complications associated with both under- and over- prescription of antibiotics. Better diagnostic techniques are required to allow a more targeted antibiotic strategy to be used.

Circulating levels of cytokines have been found to be elevated in patients with community acquired pneumonia^13–15^, aid in determining pneumonia severity^16^, and may be able to differentiate causative organisms^17^. However, findings in patients with VAP have been more equivocal^18–20^.

There is emerging evidence that oxylipins, including eicosanoids, have a role in the pathophysiology of pneumonia. Cyclooxygenase 2 is induced in human lung tissue infected with *Streptococcus Pneumoniae*^21^, and higher levels of circulating leukotriene B4 (LTB4) may predispose trauma patients to developing pulmonary complications^22^.

Measurement of inflammatory mediators within bronchoalveolar lavage fluid has been suggested as a method to exclude VAP^23^, however, measurement of circulating levels of inflammatory markers may be clinically more accepted as serum samples are routinely collected via indwelling vascular access devices, do not pose any risk to the patient and collection does not require specialist skills and so could be used as routine diagnostic tests or surveillance tools.

Clinical diagnosis of pneumonia is based on a small number of clinical parameters and studies of inflammatory markers have focussed on single or small groups of inflammatory markers with only a few studies using a panel approach to cytokine measurement^17^. We hypothesised that diagnosis of pneumonia and VAP may be aided by measuring a profile of cytokines and oxylipins in the serum of ICU patients.

## Results

### Patients

Forty five patients were recruited, 28 with brain injuries and no evidence of infection at admission to intensive care and 17 with pneumonia, one patient in the brain injury group withdrew consent. Twelve pneumonia cases were admitted from the community and five from within the hospital. Eight patients in the pneumonia group had a brain injury at or prior to inclusion on that hospital admission. Several organisms were associated with pneumonia at admission although in a large number no pathogens were identified, representing the difficulty in obtaining appropriate microbiological samples in this group of patients (Supplementary Table S1). Eight of the brain injured patients without pneumonia subsequently developed VAP, a mean of 6 days after the start of ventilation. VAP was predominantly caused by *Staphylococcus Aureus* (Supplementary Table S1). Of the remaining 19 patients one developed pneumonia but had not been ventilated for 48 h, another developed ventriculitis and a third was unable to be classified as VAP or not with either clinical pulmonary infection score (CPIS) or after independent review. For these three patients only their first, infection free, samples were used for analysis, the other 16 patients showed no clear source of infection and sequential samples were analysed. Patients were similar across the groups with respect to their demographic details and overall illness severity (Table 1). Features that differed between the pneumonia and VAP groups from those with brain injuries included markers of infection such as CRP and the higher oxygen requirement, as would be expected. Rates of antibiotic use were high even in those brain injured patients without a clear source of infection which likely represents the difficulty of diagnosis of infection amongst critically ill patients.

**Table 1:** Clinical features of included patients.

	Brain Injury (BI)	Pneumonia (P)	p-value (BI vs P)	Brain Injury Day 6 (BI6)	VAP	p-value (BI6 vs VAP)
n	27	17	—	6	8	—
Age (Mean +/− SD)	56.0 ± 16.3	53.8 ± 15.7	0.66	54.5 ± 17.1	54.4 ± 15.7	0.98
Sex, number of males (%)	15 (55.6)	11 (64.7)	0.75	3 (50.0)	5(62.5)	1.00
Ethnicity, number white European (%)	18 (66.7)	12 (70.6)	1.00	5 (83.3)	6 (75.0)	1.00
APACHE II score (Median(Range))	18 (6–31)	19 (8–31)	0.34	18.5 (11–22)^a^	16.5 (6–31)^a^	0.49
SOFA score (Mean +/− SD)	8.6 ± 2.7	10.0 ± 3.6	0.19	8.2 ± 3.3^a^	9.0 ± 2.7^a^	0.62
CPIS (Median(Range))	3 (0–6)	6 (4–7)	***1***.***5*** × ***10***^−***6***^	2.5 (1–4)	5.5 (4–9)	***0***.***001***
White cell count (109/L) (Median(Range))	11.6 (4.4–25.1)	13.8 (7.8–34.5)	0.12	10.1 (4.2–12.1)	11.0 (5.6–15.0)	0.57
C-reactive protein (mg/L) (Median(Range))	62.7 (2.8–168.4)	170.9 (34.6–325.8)	***1***.***6*** × ***10***^−***5***^	31.8 (3.7–124.8)	132.5 (37.4–311.8)	***0***.***008***
Lowest temperature (°C) (Median(Range))	36.1 (34.6–37.3)	36.1 (33.7–37.3)	0.84	36.3 (34.1–36.7)	36.5 (34.1–37.6)	0.57
Highest temperature (°C) (Median(Range))	37.5 (34.6–38.8)	37.6 (36.5–40.0)	0.18	37.7 (35.2–39.0)	38.3 (36.8–39.9)	0.41
Lowest PaO_2_:FiO_2_ (kPa) (Median(Range))	40.0 (19.1–73.8)	23.0 (9.9–43.0)	***0***.***0002***	45.4 (29.3–62.9)	25.1 (7.0–41.2)	***0***.***005***
Use of noradrenaline, n (%)	16 (59.3)	12 (70.6)	0.53	1 (16.7)	4 (50.0)	0.30
Use of antibiotics n (%)	11 (40.7)	17 (100)	***4***.***9*** × ***10***^**−*5***^	*4 (66)*	8 (100)	0.16
Enteral nutrition, n (%)	19 (70.4)	15 (88.2)	0.27	6 (100)	8 (100)	1.00
Time to sampling from start of ventilation (h) (Median(Range))	42 (9–69)	41 (22–66)	0.90	183.5 (153–204)	156.5 (78–184)	***0***.***04***

Normally distributed continuous variables are given as mean and standard deviation, non-parametric variables as median and range and categorical variables as number and percentage. P-values presented in bold text relate to parameters that were significant at the p < 0.05 level with Student’s t-test and Mann-Whitney U test for parametric and non-parametric data respectively and Fisher’s exact test for categorical data. ^a^Data taken at time of admission.

Detection rates of the 51 measured inflammatory mediators ranged from 20–100%. lipoxin A4, interleukin-6 (IL-6), interleukin-12p70, granulocyte colony stimulating factor, prostaglandin E2 (PGE2) and lipoxin B4 were undetectable in more than a third of samples and the latter three mediators were undetectable in more than 50% of samples.

### Pneumonia vs Brain Injury

**Figure 1:**
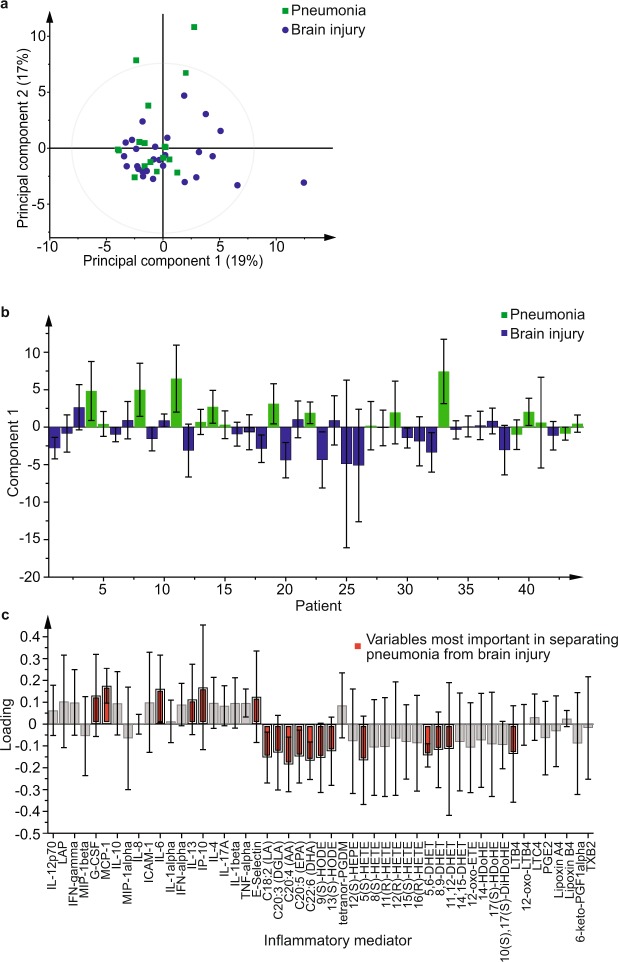
Multivariate analysis using all inflammatory mediators comparing samples taken at admission from patients admitted with pneumonia to those admitted with brain injuries. (**a**) Principal component analysis scores plot comparing principal components 1 and 2 showing pneumonia (green squares) and those admitted with brain injuries (blue circles). (**b**) Orthogonal partial least squares discriminant analysis (OPLS-DA) model with one component (R^2^Y 0.38 Q^2^ 0.22, p = 0.007) with pneumonia represented by green bars and brain injury by blue bars. Pneumonia samples project in a positive direction along the first component, y-axis, and brain injury samples in the negative direction. (**c**) Loadings plot for the model shown in b, inflammatory molecules at the top of the plot dominate in the pneumonia group and those towards the bottom in the brain injury group. Those marked in red had the greatest influence on the model. Error bars in b and c represent jack-knifed 95% standard error of the scores and loadings respectively.

Baseline samples (all taken within 70 h of admission to ICU) were compared between those with pneumonia (median time to sampling 41 h) and those with brain injury (median time to sampling 42 h). An orthogonal partial least squares discriminant analysis (OPLS-DA) model using all 51 mediators (R^2^Y 0.38, Q^2^ 0.22, p = 0.007) (Fig. 1) showed an ability to separate the pneumonia (n = 17) and brain injured (n = 27) groups with an area under the receiver operating characteristic curve (AUROC) of 0.75 (0.61–0.90). Cytokines and soluble signalling molecules were more abundant in those with pneumonia, with the exception of macrophage inflammatory protein (MIP)-1beta and MIP-1alpha, whereas the oxylipins were more abundant in those with brain injuries, with the exception of tetranor-prostaglandin D metabolite (tetranor-PGDM), leukotriene C4 and lipoxin B4 (Fig. 1). Predictive capacity of the model was improved when using only those mediators which on their own had an AUROC with a lower 95% confidence interval greater than 0.5 to differentiate brain injury from pneumonia (Supplementary Table S2) (R^2^Y 0.40, Q^2^ 0.37, p = 7.1 × 10^−5^, AUROC 0.89 (0.80–0.99)). This restricted model performed better than any individual mediator (Supplementary Table S2), white cell count (AUROC, 0.68 (0.52–0.83)) and similarly to C-reactive protein (AUROC 0.87 (0.76–0.98)). Performance was also similar to that of CPIS (AUROC 0.93 (0.86–1.0)) which had been used to help define initial group allocation.

**Figure 2:**
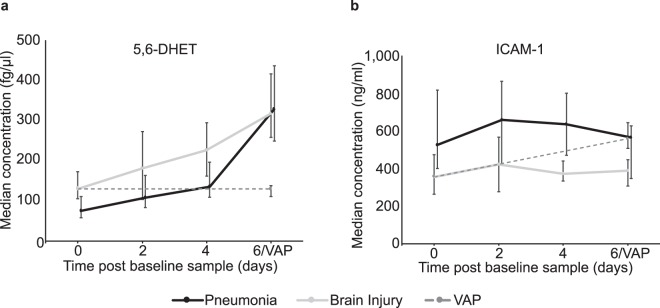
Change in concentration of 5,6-dihydroxyeicosatrienoic (5,6-DHET) (**a**) and Intercellular Adhesion Molecule-1 (ICAM-1) (**b**) over time on Intensive Care for patients with pneumonia (dark line), brain injury without infection (pale line) and brain injury who develop ventilator associated pneumonia (VAP) (dashed line). VAP represents the time that VAP was diagnosed which was not always the final time point. VAP is compared to the day 6 time point in those without infection as this was the timing of diagnosis in 50% of cases. Data are displayed as median with interquartile range.

Over the six days after ICU admission due to pneumonia, levels of 5,6-dihydroxyeicosatrienoic acid (5,6-DHET) (Fig. 2a) and 8,9-dihydroxyeicosatrienoic acid (8,9-DHET) increased with a possible increase in 5-hydroxyeicosatetraenoic acid (5-HETE) and Prostaglandin E2 (PGE2). In contrast, levels of IL-6 and 12-oxo leukotriene B4 (12-oxo-LTB4) showed a trend to decrease over time (Supplementary Fig. S1 and Table S3). Similarly, in brain injured patients who did not develop infection, 5,6-DHET (Fig. 2a) and 8,9-DHET levels increased over the duration of ICU stay and IL-6 levels fell (Supplementary Fig. S1 and Table S3).

### Ventilator Associated Pneumonia

**Figure 3:**
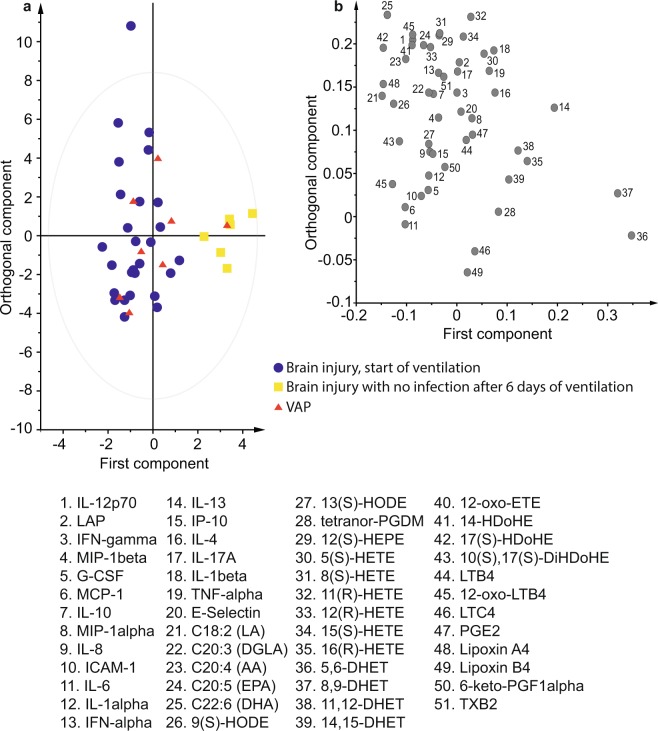
Orthogonal partial least squares discriminant analysis (OPLS-DA) comparing admission samples in patients with brain injuries to those taken after 6 days of ventilation. (**a**) OPLS-DA scores plot comparing samples taken from brain injured patients at the start of ventilation (blue circles) and after a further 6 days of ventilation for those without infection (yellow squares). Samples taken from patients with ventilator associated pneumonia have been predicted by the model to lie mainly amongst the first time point samples (red triangles). (**b**) Corresponding loadings plot for the OPLS-DA model showing the mediators that account for most of the difference between the groups. Mediators to the right of the x-axis dominate in the final time point of those without infection where as those to the far left dominate at the start of ventilation. 5,6-dihydroxyeicosatrienoic (5,6-DHET) and 8,9-dihydroxyeicosatrienoic (8,9-DHET) are the most influential oxylipins in the final time point samples.

Samples were obtained as close to the diagnosis of VAP as possible, at a median of 157 h from the start of ventilation. These were compared to similar samples from those without VAP, median time to sampling 184 h. Brain injured patients who developed VAP had a different inflammatory trajectory to those without infection. Whereas it was possible to construct an OPLS-DA model to separate samples taken from brain injured patients at admission (n = 27) from samples taken from those without infection after six days of ICU stay (n = 6) (R^2^Y 0.79, Q^2^ 0.36, p = 0.05, AUROC 0.95 (0.88–1.0)) it was not possible to separate subsequent samples from baseline in those patients who developed VAP (n = 8) (R^2^Y 0.40, Q^2^ −0.37). These patients did not exhibit the rise in 5,6-DHET (Fig. 2a) and 8,9-DHET or the fall in IL-6, that was seen with recovery from pneumonia or uncomplicated brain injury (Supplementary Fig S1 and Table S4). Soluble intercellular adhesion molecule 1 (ICAM-1) (Fig. 2b) showed a trend to increase from baseline and linoleic acid (LA) to fall in those who developed VAP, although these changes were not robust to false discovery rate detection correction. Patients who developed VAP had an inflammatory profile similar to that of brain injured patients at the time of admission (Fig. 3) despite having spent a similar number of days on the ICU.

An OPLS-DA model containing only the mediators which, based on their individual AUROCs, had independent ability to differentiate patients without infection who had spent six days on ICU (n = 6) from those who developed VAP (n = 8), differentiated these two groups well (R^2^Y 0.71, Q^2^ 0.57, p = 0.08, AUROC 0.94 (0.80–1.00)). This performed similarly to CPIS (AUROC 0.98 (0.92–1.00) and CRP (AUROC 0.91 (0.74–1.00) and retained its discriminant potential even when patients with VAP early in their stay were excluded. The mediators included in this model were 5,6-DHET, 8,9-DHET, ICAM-1, IL-6, and interleukin-8 (IL-8). OPLS-DA using all inflammatory mediators was unable to separate VAP from those without VAP.

The model containing 5,6-DHET, 8,9-DHET, ICAM-1, IL-6, and IL-8 was used in an attempt to predict VAP from samples taken 48 h and 96 h prior to those taken closest to time of VAP onset. The 48 h prior-samples were successfully classified 66% of the time whereas in the 96 h prior-samples this dropped to 48%. In both cases the majority of misclassifications were in those without VAP who were erroneously classified as VAP.

### Clinical Data

Clinical variables which had an independent ability to identify VAP from non-infected brain injured patients (Supplementary Table S5) were added to the restricted inflammatory OPLS-DA model described above. The resultant model could separate those with VAP (R^2^ 0.73, Q^2^ 0.67, p = 0.002, AUROC 0.98 (0.92–1.00)) and contained 5,6-DHET, 8,9-DHET, IL-6, IL-8, ICAM-1, platelet count, CRP and PaO_2_:FiO_2_ ratio with 5,6-DHET and platelet count being most important in the model.

## Discussion

This proof of principle study demonstrated that profiling inflammatory markers was able to differentiate patients with pneumonia from those with brain injury. Although patients with pneumonia began with relatively higher levels of cytokines at admission compared to those with brain injuries, there were similar changes in both groups over time during uncomplicated recovery. Importantly these changes, especially the increase in 5,6-DHET and 8,9-DHET and the reduction in IL-6 were not seen in brain injured patients who developed VAP. Multivariate modelling of a restricted group of mediators showed promise as a method to differentiate patients with and without VAP.

Oxylipins were the predominant mediators in brain injured patients at admission. Although the exact causes for the high levels of oxylipins in brain injury are not clear there are a number of potential explanations. Higher risk of haemorrhagic stroke has been seen in patients with high levels of polyunsaturated fatty acids in their fat composition^24^. Metabolites of 6-keto-prostaglandin F1α (6-keto-PGF1α) and thromboxane A2 have been found in the urine of patients following haemorrhagic stroke^25^. 6-keto-PGF1α represents the metabolic product of prostacyclin and may be important in the pathology of stroke by altering cerebral blood flow. The most discriminant oxylipin was 5,6-DHET which is one of the inactive metabolites of the epoxyeicosatrienoic acids (EETs) which are endogenous mediators that regulate cerebral blood flow and have protective effects against cerebral ischaemia^26^. Levels of EETs and their metabolites^27,28^ have been found to be altered in patients who have suffered from stroke compared to healthy controls and species of EETs have been implicated in the pathogenesis of stroke, cerebral haemorrhage and traumatic brain injury^29^.

All cytokines tended to be increased on admission in those with pneumonia compared to brain injury. We observed E-selectin, MCP-1, ICAM-1, and interferon gamma-induced protein 10 (IP-10) to be most able to differentiate pneumonia from brain injury. These findings matched the elevated concentrations of these mediators previously described in pneumonia^17,30,31^.

Over the course of ICU stay, levels of 5,6-DHET and 8,9-DHET increased in both those with brain injuries and those with pneumonia, which mirrors findings seen between the early and late post-operative periods following surgery^32^, which may reflect the anti-inflammatory properties of the precursor EETs and their role modulating pulmonary inflammation^33^. Patients who developed VAP also showed a trend to increased levels of the pro-inflammatory mediators IL-6, IL-8 and ICAM-1, reflecting an inflammatory response to infection. Multivariate models using the mediators that showed different trajectories performed well at identifying those with VAP. Although a panel of inflammatory mediators were measured using a combination of analytical platforms in this study we have demonstrated that only a subset of these may be needed to aid diagnosis. As such, clinical application could use a small subset of biomarkers which may be measured using less technically demanding processes. Models may be improved when clinical data is added, however, caution is needed as the initial diagnosis of VAP was based on clinical parameters.

Despite promising findings there are limitations to this study. Firstly, the small number of patients limit the interpretation of the multivariate models and the possibility of over-fitting must be considered. The optimal test of the models would be with a validation set of data, however, the low numbers prevented this. Many patients recruited with pneumonia also had concomitant brain injuries potentially leading to overlap between the conditions. No true “gold standard” tests exist by which to diagnose VAP and a number of diagnostic criteria have been suggested. In this study we applied the CPIS score, however, we recognise that this has its limitations and its accuracy has been questioned^34^. Antibiotic use was high even in those assessed not to have infection, representing the great difficulty in this group of critically ill patients of making this diagnosis. For these reasons every effort was made to ensure correct classification of patients using a combination of bedside clinical opinion, objective CPIS and independent second assessment. However, these methods used to ensure defined study groups may lead to selection bias, with borderline cases excluded. In this pilot study it was important to understand the potential for these biomarkers to aid diagnosis where uncertainty was minimised. Further work will be needed to assess their use in the clinical environment where diagnosis may not be so well defined and other infections may be present. Early discharges and deaths can complicate analysing samples over time as both drop out of analysis. Further work looking at different cohorts of intensive care patients is needed to test the generalisability of these results and to test the specificity of the models to diagnose VAP compared with other ICU acquired infections.

## Conclusion

Measurement of a panel of inflammatory mediators including oxylipins, cytokines and soluble adhesion molecules showed ability to differentiate critically ill patients with pneumonia from patients with brain injuries, and brain injured patients who developed VAP from those who did not. Measurement of a small number of these mediators may be useful clinical tests especially in combination with clinical data to aid the diagnosis of VAP.

## Methods

### Study Participants

Patients were recruited from Imperial College Healthcare NHS Trust, London, following independent ethics committee approval (North London REC 10/H0709/77) and in accordance with the standards indicated by the Declaration of Helsinki. Patients who had systemic inflammation (2/4 SIRS criteria^35^), were receiving mechanical ventilation and expected to require ventilation for more than 48 hours were eligible for inclusion. Patients who were immunosuppressed, were receiving granulocyte colony stimulating factor, were under 16 yrs or whose next of kin refused assent for inclusion into the study were excluded. Two groups of patients were enrolled; the first had a primary diagnosis of brain injury with no evidence of pneumonia, including subarachnoid haemorrhage, cerebrovascular accident, isolated head injury, status epilepticus or primary brain tumour. The second was composed of patients who had pneumonia. Diagnosis of pneumonia was based on the opinion of the primary physician, which took into account clinical history, examination, laboratory and radiology results. However, as clinical assessment is subjective and known to have a low level of accuracy this group was further refined by the application of the clinical pulmonary infection score (CPIS)^36^ as described previously^37^. The use of this score allowed an objective approach to be taken to the diagnosis of pneumonia. When there was either a borderline CPIS score or clinical doubt as to the diagnosis, these cases were reviewed by an independent clinical assessor. Brain injury was selected as the control group as these patients are critically ill and often require a period of prolonged ventilation, without having infection at the point of enrolment. This group has a significant risk of developing VAP providing the opportunity to prospectively acquire samples from patients developing VAP without the confounding of other infections.

Enrolment occurred within 48 h of ICU admission. As the recruited patients were either sedated, to facilitate mechanical ventilation, or were unconscious, due to a brain injury, ethics approval allowed initial written, informed assent for enrolment to be obtained from the next of kin, with retrospective written, informed consent for inclusion in the study being obtained from the patient on recovery. Blood samples were collected as soon after enrolment as possible and then at 48 h intervals, until either the patient left the ICU or four samples had been collected. Serum was separated within 45 minutes and then stored immediately at −80 °C prior to batch analysis.

Patients with brain injuries were followed up daily and VAP was diagnosed in those who had been ventilated for >48 h in whom the CPIS was greater than 6. As band form quantification and Gram staining of tracheal aspirates were not routinely performed at our institution, some patients had equivocal CPIS. Patients with scores of 5 or 6 were assessed by an independent clinician, blinded to the inflammatory mediator results, and classified as pneumonia, VAP or no VAP. As this was an exploratory study no power calculation could be performed to determine the number of participants needed. We aimed to recruit six patients to each group. Given an assumed VAP rate of 25% this meant that we needed to recruit at least 24 patients in the brain injured group.

### Clinical Variables

A comprehensive set of clinical data were recorded for each day of a patient’s ICU stay. Data were collected at 8:00am every morning with minimum and maximum values recorded for the previous 24 h period. There was no imputation for missing data. All data were checked manually to ensure values fell within physiological ranges.

### Oxylipins

Oxylipin levels were measured using liquid chromatography coupled to tandem mass spectrometric detection (LC-MS/MS) using a targeted method developed internally for the Division of Computational and Systems Medicine of Imperial College London^38^. It was designed to quantify up to 48 oxylipins using solid phase extraction of the analytes from serum. Of the 48 oxylipins targeted 31 were quantifiable within this patient cohort (Supplementary Table S6). Oxylipin concentrations that fell below the lower limit of quantification were recorded as zero.

### Cytokines

A panel of human inflammatory cytokines were measured using the commercially available 20plex FlowCytomix™ kit (eBioscience, San Diego, USA). Samples were processed following the manufacturer’s instructions. The kit allowed 20 cytokines to be measured using flow cytometry (Supplementary Table S6), by utilising two bead populations of differing sizes. Data files were exported into FlowCytomix Pro™ software (eBioscience, San Diego, USA) and cytokine concentrations were calculated by comparing the mean fluorescence intensity of each sample to the standard curves. Samples that saturated the concentration curves were rerun using a ten-fold dilution and concentrations calculated from this result. Where the calculated concentration from the repeated samples failed to exceed the upper limit of the standard curve, the average was taken from the repeated value and the upper limit of quantification from the standard curve. Where cytokine concentrations fell below the level of detection a value of zero was assigned. In line with the manufacturer’s instructions samples were run in duplicate and the cytokine concentration was based on the average of the two measurements. Where there was discrepancy between the duplicates samples were re-run.

### Comparisons

1. To identify inflammatory markers that would help identify pneumonia at ICU admission, those categorised as pneumonia were compared to those admitted with brain injury without infection.
2. To identify differences in inflammatory trajectories the admission samples were compared to day-6 samples collected in those with pneumonia and those brain injured patients who did not develop VAP.
3. To identify biomarkers that may help diagnose VAP, patients with VAP were compared to a group who had been ventilated for a similar length of time but who had not developed infection. This was determined to be day-6 on ICU based on the most frequent time point of sampling in those with VAP. The latter comparison was chosen to ensure that as far as possible similar patients were being compared and any potential confounding due to duration of ventilation was eliminated.

### Statistical analysis

Routine data handling was performed in Excel (Microsoft, USA). Mediator concentrations were scaled to unit variance, for multivariate analysis, to address the exaggerated influence of analytes with naturally higher variance. Initial exploration with principal component analysis (PCA) looked for natural clustering and outliers before supervised analysis using orthogonal partial least squares discriminant analysis (OPLS-DA) was used to generate models to optimally separate predefined groups. OPLS-DA models were cross validated using seven fold cross-validation using a “leave-many-out” methodology. Important variables were identified by examining the loadings and variable importance (VIP) scores associated with each model. Multivariate analysis was performed using the SIMCA 14.0 statistical package (Umetrics, Sweden). To assess the reliability of the models a cross-validated analysis of variance was used (CV-ANOVA)^39^. Permutation testing was also performed where the Y variables were randomly generated 999 times, in order to scramble the true class information, and a new model constructed for each permutation. The *Q*^2^ and *R*^2^ could then be compared with those generated from the random models. Predictive capacity of OPLS-DA models was assessed with receiver operating characteristic curves based on cross validated scores. Univariate comparisons were made using the Student’s t-test for normally distributed continuous variables, the Mann Whitney U test for non-parametric variables and the Fisher’s exact test for categorical variables, two tailed tests were used in all cases. The Kolmogorov-Smirnov test was used to assess normality. The Benjamini Hochberg procedure to correct for false discovery rate (FDR) was used when multiple comparisons were made. Discriminant ability of individual mediators was assessed with the area under the receiver operating characteristic curves (AUROC). A significant p-value was taken as p < 0.05. Univariate analysis was performed with SPSS version 24 (IBM, USA) and R (R Foundation^40^). To illustrate trajectories of important mediators medians and interquartile ranges were plotted for each sampling time point.

## Electronic supplementary material

Supplementary Appendix

## Data Availability

The datasets generated and analysed during the current study are available from the corresponding author on submission of a data request application.
