# Supplementary material for: Profiling inflammatory markers in patients with pneumonia on intensive care
Source: Sci Rep. 2018 Oct 3;8:14736. doi: 10.1038/s41598-018-32938-6 (PMC6170441; doi:10.1038/s41598-018-32938-6)
Supplement: Supplementary file 1 — Supplementary Appendix [file 41598_2018_32938_MOESM1_ESM.pdf]

Profiling inflammatory markers in patients with pneumonia on intensive care
Supplementary Appendix

David B Antcliffe PhD, Arnaud M Wolfer PhD, Kieran P O'Dea PhD, Masao Takata PhD, Elaine Holmes PhD, Anthony C Gordon MD

Results

Supplementary Table S1. Microorganisms isolated in patients with pneumonia and VAP, it was possible for patients to have more than one organism.

Organism	***Pneumonia (n)***	***VAP (n)***
Total Patients	*17*	*8*
No Growth	*8*	*2*
Streptococcus Pneumoniae	*2*	*0*
Pseudomonas	*2*	*0*
Staphylococcus Aureus	*2*	*4*
Enterobacter Cloacae	*1*	*0*
Enterobacter Ludwigii	*1*	*0*
Escherichia Coli	*1*	*0*
Moraxella Catarrhalis	*1*	*0*
Serratia Marcescens	*1*	*1*
Morganella Morganii	*1*	*0*
Klebsiella Pneumoniae	*0*	*1*

Supplementary Table S2. Predictive capacity of individual inflammatory mediators to separate pneumonia from brain injury at the time of admission. (AUROC, area under the receiver operating characteristic curve, 95% CI, 95% confidence interval for the AUROC)

Mediator	AUROC	95% CI	p-value
5,6-DHET	0.806	0.652-0.96	***0.001***
C22:6 (DHA)	0.773	0.623-0.924	***0.002***
6-keto-PGF1α	0.765	0.624-0.906	***0.003***
E-Selectin	0.771	0.626-0.916	***0.003***
IP-10	0.708	0.545-0.872	***0.021***
MCP-1	0.702	0.535-0.868	***0.026***
C20:5 (EPA)	0.695	0.529-0.861	***0.031***
13(S)-HODE	0.693	0.537-0.849	***0.033***
ICAM-1	0.691	0.521-0.86	***0.035***
9(S)-HODE	0.684	0.528-0.84	***0.042***
Lipoxin B4	0.662	0.49-0.835	0.073
LTB4	0.651	0.482-0.821	0.094
5(S)-HETE	0.644	0.476-0.811	0.112
IL-6	0.634	0.448-0.82	0.138
8,9-DHET	0.631	0.462-0.799	0.148
12(R)-HETE	0.63	0.454-0.805	0.152
C18:2 (LA)	0.627	0.455-0.799	0.159
tetranor-PGDM	0.625	0.457-0.794	0.166
MIP-1α	0.621	0.456-0.786	0.181
C20:4 (AA)	0.619	0.447-0.791	0.189
TXB2	0.619	0.441-0.796	0.189
14,15-DHET	0.615	0.441-0.79	0.201
IL-13	0.606	0.432-0.78	0.242
11,12-DHET	0.599	0.423-0.776	0.273
12-oxo-ETE	0.596	0.42-0.772	0.289
IL-10	0.592	0.418-0.765	0.311
TNFα	0.582	0.397-0.766	0.366
IFNα	0.576	0.401-0.752	0.399
14-HDoHE	0.569	0.388-0.749	0.448
IL-17A	0.568	0.389-0.746	0.455
PGE2	0.566	0.393-0.739	0.462
Lipoxin A4	0.562	0.386-0.738	0.492
10(S),17(S)-DiHDoHE	0.557	0.384-0.729	0.531
11(R)-HETE	0.556	0.381-0.73	0.539
IL-1β	0.551	0.365-0.737	0.571
16(R)-HETE	0.546	0.368-0.723	0.613
G-CSF	0.545	0.365-0.724	0.621
MIP-1β	0.54	0.363-0.718	0.656
12-oxo-LTB4	0.54	0.356-0.724	0.656
8(S)-HETE	0.538	0.36-0.716	0.673
12(S)-HEPE	0.536	0.356-0.716	0.691
17(S)-HDoHE	0.532	0.358-0.705	0.727
IL-12p70	0.529	0.353-0.706	0.745
IL-4	0.527	0.344-0.711	0.763
IL-8	0.512	0.339-0.685	0.895
IFNγ	0.51	0.328-0.692	0.914
C20:3 (DGLA)	0.507	0.332-0.682	0.942
LTC4	0.505	0.324-0.687	0.952

LAP	0.503	0.313-0.694	0.971
15(S)-HETE	0.501	0.323-0.679	0.99
IL-1α	0.5	0.322-0.678	1

Supplementary Table S3. Comparison of inflammatory mediator concentrations between the first and last samples collected for those with pneumonia and brain injury. Concentrations are given as median and range. (p-value from two tailed Mann-Whitney U test, q-values are given after FDR correction)

**Mediator**	**Brain injury baseline sample (TP1)**	**Brain injury 6 days after baseline sample (TP4)**	**Brain Injury TP1 vs TP4**		**Pneumonia baseline sample (TP1)**	**Pneumonia 6 days after baseline sample (TP4)**	**Pneumonia TP1 vs TP4**	
n	27	6	**p-value**	**q-value**	17	8	**p-value**	**q-value**
IL-12p70 (pg/ml)	2.8(0-41.5)	0.4(0-18.2)	0.324	0.87	13.7(0-48.0)	16.1(0-31.0)	0.977	1.00
LAP (ng/ml)	10.1(0-48.5)	14.3(10.0-18.3)	0.222	0.81	9.7(2.5-199.1)	7.4(0.9-13.8)	0.475	0.76
IFNγ (pg/ml)	67.4(0-623.8)	80.3(21.4-105.4)	0.874	0.91	56.4(0-1216.9)	44.8(5.9-174.6)	0.754	0.84
MIP-1β (pg/ml)	144.0(45.0-13,259.6)	143.7(59.0-1,350.8)	0.91	0.91	199.7(33.3-2,177.8)	106.3(47.5-644.7)	0.588	0.77
G-CSF (pg/ml)	0(0-917.3)	0(0-8.6)	0.733	0.91	0(0-1,342.0)	0(0-64.5)	0.475	0.76
MCP-1 (pg/ml)	289.5(102.8-11,90.5)	207.1(156.3-354.7)	0.424	0.91	469.1(95.8-4324.2)	360.4(98.6-2,003.7)	0.475	0.76
IL-10 (pg/ml)	4.6(0-33.8)	1.5(0-26.1)	0.803	0.91	5.3(0-86.2)	5.3(0-47.9)	0.475	0.76
MIP-1α (pg/ml)	58.8(0.5-6,845.9)	111.2(20.4-3,045.2)	0.398	0.91	95.5(7.8-904.0)	64.0(26.2-255.7)	0.315	0.76
IL-8 (pg/ml)	192.4(0-15,943.3)	37.0(21.0-9,105.3)	0.324	0.87	192.1(4.4-14,407.0)	157.3(39.9-2,652.5)	0.628	0.78
ICAM-1 (ng/ml)	357.5(111.0-2,079.0)	390.1(68.6-474.2)	0.874	0.91	526.4(87.5-3,162.5)	567.2(91.7-1,036.6)	0.549	0.77
IL-6 (pg/ml)	11.1(0-401.5)	0(0-2.0)	0.002	***0.03***	53.4(0-429.1)	0(0-54.9)	0.007	0.09
IL-1α (pg/ml)	39.9(0-3099.5)	4.8(0-1084.8)	0.324	0.87	34.9(0-4243.1)	26.5(0.4-444.6)	0.511	0.77
IFNα (pg/ml)	80.9(0-563.1)	44.3(0-167.3)	0.451	0.91	113.2(0-643.2)	60.2(0-271.1)	0.374	0.76
IL-13 (pg/ml)	109.8(0-202.7)	146.3(89.7-194.8)	0.051	0.65	110.1(66.4-506.9)	109.4(80.8-136.0)	0.588	0.77
IP-10 (pg/ml)	68.3(31.0-215.0)	56.2(41.0-163.4)	0.838	0.91	115.7(37.1-2,508.8)	99.6(38.0-2,479.7)	0.711	0.84
IL-4 (pg/ml)	45.4(0-177.4)	55.3(36.9-66.1)	0.348	0.89	47.3(0-168.8)	35.4(15.9-78.2)	0.754	0.84
IL-17A (pg/ml)	26.3(0-496.7)	60.1(8.4-106.6)	0.508	0.91	63.3(0-451.0)	11.9(0-143.2)	0.262	0.76
IL-1β (pg/ml)	82.9(0-240.1)	103.8(39.2-162.3)	0.569	0.91	100.4(0-416.3)	45.5(13.9-141.5)	0.549	0.77
TNFα (pg/ml)	8.3(0-123.7)	19.8(8.2-50.4)	0.132	0.80	24.1(0-69.8)	5.1(0-35.5)	0.406	0.76
E-Selectin (ng/ml)	75.0(30.8-292.7)	61.3(34.8-180.5)	0.633	0.91	160.9(46.0-618.5)	118.9(45.6-373.6)	0.262	0.76
C18:2 (LA) (ng/ul)	18.5(5.6-46.3)	13.8(4.5-23.1)	0.189	0.80	13.9(4.0-30.1)	9.1(2.8-20.9)	0.124	0.66
C20:3 (DGLA) (pg/ul)	191.1(60.3-447.4)	210.0(64.9-283.4)	0.803	0.91	187.1(95.7-355.2)	196.3(127.5-337.8)	0.628	0.78
C20:4 (AA) (ng/ul)	1.1(0.6-2.8)	1.1(0.4-1.4)	0.451	0.91	0.9(0.4 -1.8)	0.8(0.4-1.6)	0.406	0.76
C20:5 (EPA) (pg/ul)	410.8 (147.8-2,065.7)	402.3(149.9-544.2)	0.699	0.91	249.7(84.1-1,053.3)	264.2(94.2-959.1)	0.754	0.84
C22:6 (DHA) (ng/ul)	1.2(0.4-3.7)	0.8(0.4-1.3)	0.098	0.71	0.6(0.1-2.6)	0.5(0.2-1.7)	0.344	0.76
9(S)-HODE (pg/ul)	4.4(1.1-19.9)	2.9(1.8-6.3)	0.189	0.80	3.1 (0.8-6.9)	2.7(1.2-6.5)	0.887	0.94

13(S)-HODE (pg/ul)	5.5(1.2-24.2)	4.8(2.1-12.7)	0.768	0.91	4.2(0.9-10.8)	3.2(1.5-6.0)	0.932	0.97
tetranor-PGDM (pg/ul)	3.7(0-8.8)	5.3(3.0-6.8)	0.205	0.80	4.4(2.8-17.9)	8.8(3.2-53.3)	0.175	0.66
12(S)-HEPE (fg/ul)	920.6(489.3-3,791.0)	919.7(302.8-1,516.5)	0.633	0.91	930.0(349.5-2,489.1)	827.6(304.2-1,994.2)	0.842	0.91
5(S)-HETE (fg/ul)	573.0(0-1,935.2)	743.2(497.9-1,048.0)	0.173	0.80	491.1(0-991.4)	944.2(261.8-1,538.4)	0.003	0.05
8(S)-HETE (fg/ul)	168.3(56.3-1,264.0)	137.8(106.4-409.2)	0.733	0.91	166.2(48.1-610.3)	209.9(46.0-503.6)	0.475	0.76
11(R)-HETE (fg/ul)	190.6(0-989.1)	204.4(130.1-636.2)	0.6	0.91	184.0(0-958.5)	319.0(114.8-1,551.2)	0.14	0.66
12(R)-HETE (pg/ul)	2.6(0.2-69.1)	1.9(0.5-11.5)	0.633	0.91	0.9(0.3-34.0)	1.7(0.4-29.1)	1.00	1.00
15(S)-HETE (fg/ul)	414.2(122.9-1,722.2)	563.6(258.0-1,442.6)	0.768	0.91	441.8(117.7-1,196.5)	901.3(285.6-2,090.8)	0.157	0.66
16(R)-HETE (fg/ul)	201.3(83.0-564.8)	204.2(157.0-636.9)	0.398	0.91	175.4(93.2-375.1)	200.9(122.4-424.5)	0.374	0.76
5,6-DHET (fg/ul)	130.2(64.7-264.7)	316.7(208.7-440.8)	0.0004	***0.01***	74.2(38.9-231.3)	327.9(187.9-590.3)	$1.3x10^{-5}$	***0.0007***
8,9-DHET (fg/ul)	102.3(49.7-212.0)	220.3(157.2-379.5)	0.0001	***0.01***	89.7(51.7-180.3)	211.3(109.1-409.3)	0.0003	***0.01***
11,12-DHET (fg/ul)	175.3(60.3-468.1)	198.4(94.4-805.0)	0.733	0.91	158.9(60.4-381.7)	213.2(105.2-415.4)	0.175	0.66
14,15-DHET (fg/ul)	240.0(107.2-632.6)	212.1(163.7-1,274.7)	0.803	0.91	201.6(95.0-488.6)	242.1(129.5-672.7)	0.475	0.76
12-oxo-ETE (fg/ul)	218.4(0-1,671.9)	183.7(0-365.5)	0.324	0.87	174.4(0-568.0)	216.75(0-512.3)	0.374	0.76
14-HDoHE (pg/ul)	1.2(0.3-10.9)	0.8(0.4-1.2)	0.26	0.87	0.8(0.3-3.7)	0.7(0.3-1.3)	0.588	0.77
17(S)-HDoHE (fg/ul)	691.4(312.6-2,343.8)	598.3(0-765.5)	0.173	0.80	652.7(337.2-1,860.8)	546.5(253.9-2,248.1)	0.374	0.76
10(S),17(S)-DiHDoHE (fg/ul)	12.4(5.4-41.2)	12.7(3.4-14.7)	0.538	0.91	11.0(4.4-23.0)	12.1(6.7-23.4)	0.711	0.84
LTB4 (fg/ul)	598.3(243.8-1,572.9)	628.8(352.2-1,475.6)	0.768	0.91	554.2(150.9-1,004.7)	618.5(336.4-1,349.7)	0.175	0.66
12-oxo-LTB4 (pg/ul)	11.9(3.4-111.3)	6.2(4.2-15.3)	0.064	0.65	13.9(1.9-122.4)	5.0(2.4-12.1)	0.037	0.31
LTC4 (fg/ul)	293.4(0-788.3)	265.7(0-638.3)	0.874	0.91	242.6(0-1,040.5)	290.7(128.9-725.2)	0.511	0.77
PGE2 (fg/ul)	0(0-562.7)	0(0-634.6)	0.91	0.91	0(0-453.7)	161.3(0-550.9)	0.031	0.31
Lipoxin A4 (fg/ul)	198.9(0-2,300.6)	0(0-218.0)	0.098	0.71	208.0(0-3,648.1)	55.0(0-1,539.7)	0.11	0.66
Lipoxin B4 (fg/ul)	0(0-2,474.3)	0(0-1,636.6)	0.838	0.91	0(0-2,430.0)	0(0-638.9)	0.194	0.66
6-keto-PGF1α (pg/ul)	2.3(0-12.5)	1.7(0.6-5.8)	0.569	0.91	0.9(0-4.2)	0.6(0-3.3)	0.475	0.76
TXB2 (pg/ul)	0.6(0-10.9)	0.8(0-2.7)	0.838	0.91	0(0-25.7)	0.7(0-44.5)	0.194	0.66

Supplementary Table S4. Comparison of inflammatory mediator levels between the first samples collected from those with brain injury compared to those samples taken at the time of VAP diagnosis. Concentrations are given as median and range. (p-value from Mann-Whitney U test, q-values are given after FDR correction)

Mediator	Brain injury baseline sample	Time of VAP diagnosis	p-value	q-value
n	27	8	-	-
ICAM-1 (ng/ml)	357.5(111.0-2,079.0)	558.4 (326.8-2,430.7)	0.01	0.51
C18:2 (LA) (ng/ul)	18.5(5.6-46.3)	12.9(5.0-20.1)	0.04	0.97
IP-10 (pg/ml)	68.3(31.0-215.0)	122.5 (48.7-570.7)	0.06	0.97
C22:6 (DHA) (ng/ul)	1.2(0.4-3.7)	0.9(0.4-1.5)	0.11	0.97
9(S)-HODE (pg/ul)	4.4(1.1-19.9)	3.2(1.8-6.0)	0.13	0.97
MCP-1 (pg/ml)	289.5(102.8-1190.5)	408.4(148.9-668.5)	0.22	0.97
TNFα (pg/ml)	8.3(0-123.7)	22.0 (0-63.8)	0.27	0.97
LTC4 (fg/ul)	293.4(0-788.3)	400.8(0-777.1)	0.27	0.97
6-keto-PGF1α (pg/ul)	2.3(0-12.5)	1.4(0-6.6)	0.27	0.97
IL-12p70 (pg/ml)	2.8(0-41.5)	18.2 (0-31.7)	0.32	0.97
IL-8 (pg/ml)	192.4(0-15,943.3)	872.6 (57.8-11,083.6)	0.32	0.97
tetranor-PGDM (pg/ul)	3.7(0-8.8)	5.0 (2.5-12.2)	0.32	0.97
IL-1α (pg/ml)	39.9(0-3,099.5)	171.8 (5.3-1,097.3)	0.36	0.97
E-Selectin (ng/ml)	75.0(30.8-292.7)	86.6(55.0-233.1)	0.36	0.97
10(S),17(S)-DiHDoHE (fg/ul)	12.4(5.4-41.2)	9.9(3.5-26.7)	0.36	0.97
MIP-1β (pg/ml)	144.0(45.0-13,259.6)	410.4(90.4-3,830.2)	0.38	0.97
IL-17A (pg/ml)	26.3(0-496.7)	76.3(0-217.6)	0.45	0.97
11(R)-HETE (fg/ul)	190.6(0-989.1)	240.7(109.9-392.0)	0.45	0.97
IL-13 (pg/ml)	109.8(0-202.7)	112.0(36.2-201.8)	0.5	0.97
LAP (ng/ml)	10.1(0-48.5)	8.5(0-43.5)	0.55	0.97
13(S)-HODE (pg/ul)	5.5(1.2-24.2)	4.6(2.2-11.1)	0.55	0.97
17(S)-HDoHE (fg/ul)	691.4(312.6-2,343.8)	700.0(383.6-915.4)	0.55	0.97
12(S)-HEPE (fg/ul)	920.6(489.3-3791.0)	857.6(454.7-1,133.8)	0.58	0.97
5(S)-HETE (fg/ul)	573.0(0-1935.2)	671.2(348.3-1,454.6)	0.58	0.97
16(R)-HETE (fg/ul)	201.3(83.0-564.8)	146.2(62.4-635.2)	0.6	0.97
IFNγ (pg/ml)	67.4(0-623.8)	98.4(0-204.6)	0.63	0.97
IL-10 (pg/ml)	4.6(0-33.8)	11.5(0-33.6)	0.63	0.97
C20:5 (EPA) (pg/ul)	410.8(147.8-2,065.7)	381.2(212.1-541.0)	0.63	0.97
LTB4 (fg/ul)	598.3(243.8-1,572.9)	708.0(325.9-1,297.6)	0.63	0.97
Lipoxin A4 (fg/ul)	198.9(0-2,300.6)	357.7(0-2,668.5)	0.63	0.97
15(S)-HETE (fg/ul)	414.2(122.9-1,722.2)	501.2(293.9-723.1)	0.66	0.97
8(S)-HETE (fg/ul)	168.3(56.3-1,264)	207.7(70.1-451.5)	0.69	0.97
MIP-1α (pg/ml)	58.8(0.5-6,845.9)	50.5(13.7-526.9)	0.71	0.97
14-HDoHE (pg/ul)	1.2(0.3-10.9)	0.9(0.4-3.0)	0.74	0.97
IL-6 (pg/ml)	11.1(0-401.5)	15.7(0-43.0)	0.77	0.97
IL-4 (pg/ml)	45.4(0-177.4)	44.2(0-107.2)	0.77	0.97
IL-1β (pg/ml)	82.9(0-240.1)	77.1(0-193.4)	0.77	0.97
14,15-DHET (fg/ul)	240(107.2-632.6)	240.2(120.6-938.5)	0.77	0.97
12-oxo-LTB4 (pg/ul)	11.9(3.4-111.3)	13.9(3.9-65.0)	0.8	0.97
IFNα (pg/ml)	80.9(0-563.1)	65.4(0-327.1)	0.83	0.97
5,6-DHET (fg/ul)	130.2(64.7-264.7)	130.1(74.1-296.9)	0.83	0.97
11,12-DHET (fg/ul)	175.3(60.3-468.1)	168.2 (90.8-511.8)	0.86	0.97
12(R)-HETE (pg/ul)	2.6(0.2-69.1)	2.9 (0.7-5.7)	0.89	0.97
8,9-DHET (fg/ul)	102.3(49.7-212.0)	87.3 (73.6-348.5)	0.89	0.97
G-CSF (pg/ml)	0(0-917.3)	0 (0-188.9)	0.89	0.97
C20:3 (DGLA) (pg/ul)	191.1(60.3-447.4)	204.7(134.7-269.9)	0.92	0.97

C20:4 (AA) (ng/ul)	1.1(0.6-2.8)	1.2(0.7-1.8)	0.92	0.97
12-oxo-ETE (fg/ul)	218.4(0-1671.9)	186.0(128.5-475.4)	0.95	0.97
PGE2 (fg/ul)	0(0-562.7)	55.5(0-195.6)	0.95	0.97
Lipoxin B4 (fg/ul)	0(0-2474.3)	0 (0-887.9)	0.95	0.97
TXB2(pg/ul)	0.6(0-10.9)	0.8 (0-4.6)	0.99	0.99

Supplementary Table S5. Predictive capacity of clinical variables to separate ventilator associated pneumonia (VAP) from brain injury after a similar duration of ventilation. (AUROC, area under the receiver operating characteristic curve, 95% CI, 95% confidence interval for the AUROC). [a]*Data missing from one patient with VAP.* [b]Data missing from one patient with brain injury.

Clinical Data	AUROC	95% CI	p-value	Direction of change in VAP
Lowest PaO_2:FiO_2	0.938	0.814-1	***0.007***	↓
[a]Platelet count	0.929	0.776-1	***0.01***	↓
[a]C-reactive protein	0.905	0.737-1	***0.015***	↑
Highest PaO2:FiO2	0.813	0.567-1	0.053	↓
Highest systolic blood pressure	0.771	0.504-1	0.093	↑
Highest $PaCO_2$	0.75	0.46-1	0.121	↑
Highest base excess	0.75	0.435-1	0.121	↑
[a]Creatinine	0.75	0.472-1	0.134	↑
Lowest pH	0.74	0.469-1	0.138	↓
Highest bicarbonate	0.74	0.426-1	0.138	↑
[a]Fibrinogen	0.738	0.453-1	0.153	↑
Lowest heart rate	0.729	0.448-1	0.156	↓
Highest lactate	0.719	0.394-1	0.175	↑
Lowest GCS	0.708	0.423-0.994	0.197	↓
[a]Sodium	0.714	0.378-1	0.199	↑
Lowest diastolic blood pressure	0.698	0.402-0.994	0.22	↓
[a b]Bilirubin	0.714	0.406-1	0.223	↑
[a]Activated partial thromboplastin time	0.702	0.384-1	0.225	↑
[a]Potassium	0.702	0.406-0.999	0.225	↓
[a]Chloride	0.69	0.364-1	0.253	↑
Highest GCS	0.677	0.386-0.969	0.272	↓
Lowest mean arterial pressure	0.677	0.384-0.971	0.272	↓
[a]Prothrombin time	0.679	0.371-0.986	0.284	↓
[a b]Alkaline Phosphatase	0.686	0.375-0.996	0.291	↑
[a]White cell count	0.667	0.357-0.976	0.317	↑
[a]Highest glucose	0.646	0.347-0.945	0.366	↑
Highest heart rate	0.635	0.336-0.935	0.401	↓
Highest temperature	0.635	0.335-0.935	0.401	↑
Highest diastolic blood pressure	0.625	0.324-0.926	0.439	↑
Lowest lactate	0.625	0.307-0.943	0.439	↑
[a b]Phosphate	0.614	0.266-0.962	0.516	↓
Lowest temperature	0.604	0.294-0.915	0.519	↑
[a]Magnesium	0.595	0.255-0.935	0.568	↑
Highest mean arterial pressure	0.583	0.273-0.894	0.606	↑
Lowest bicarbonate	0.583	0.26-0.907	0.606	↑
Lowest Systolic blood pressure	0.573	0.26-0.886	0.651	↓
Lowest base excess	0.573	0.243-0.903	0.651	↑
Highest pH	0.563	0.247-0.878	0.699	↑
[a]Urea	0.56	0.221-0.898	0.721	↑
Lowest $PaCO_2$	0.552	0.229-0.875	0.747	↑

[a]Haematocrit	0.536	0.202-0.869	0.83	↑
[a b]Alanine transaminase	0.471	0.131-0.811	0.871	↑
[a]Corrected Calcium	0.529	0.167-0.89	0.871	↑
[a]Haemoglobin	0.512	0.174-0.849	0.943	↑
Lowest Glucose	0.51	0.184-0.837	0.949	↑
[a b]Albumin	0.5	0.16-0.84	1	↑

Supplementary Table S6. Inflammatory mediators measured, those in italics are those that were unquantifiable in all samples.

Oxylipins	Cytokines and soluble signalling molecules
**C18:2 (LA)**	**G-CSF**
**C20:3 (DGLA)**	**ICAM-1**
**C20:4 (AA)**	**IFNγ**
**C20:5 (EPA)**	**IL-6**
**C22:6 (DHA)**	**IL-8**
**9(S)-HODE**	**IL-10**
**13(S)-HODE**	**IL12p70**
**Tetranor-PGDM**	**LAP**
**12(S)-HEPE**	**MCP-1**
**5(S)-HETE**	**MIP1α**
**8(S)-HETE**	**MIP1ß**
**11(R)-HETE**	**E-Selectin**
**12(R)-HETE**	**IFNα**
**15(S)-HETE**	**IL-1α**
**16(R)-HETE**	**IL-1ß**
**5,6-DHET**	**IL-4**
**8,9-DHET**	**IL-13**
**11,12-DHET**	**IL-17A**
**14,15-DHET**	**IP-10**
**12-oxo-ETE**	**TNFα**
**14-HDoHE**	
**17(S)-HDoHE**	
**10(S),17(S)-DiHDoHE**	
**LTB4**	
**12-oxo-LTB4**	
**LTC4**	
**PGE2**	
**Lipoxin A4**	
**Lipoxin B4**	
**6-keto-PGF1 α**	
**TXB2**	
Tetranor-PGEM	
Tetranor-PGFM	
15(S)-HEPE	
5,6-EET	
8,9-EET	
11,12-EET	
14,15-EET	
5-oxo-ETE	
LTD4	
LTE4	
PGD2	
PGF2α	
8-iso-PGF2α	
15dPGJ2	
11-dehydro TXB2	
Resolvin D1	
Resolvin D2	

Supplementary Figure S1. Change in concentration of 8,9-dihydroxyeicosatrienoic (8,9-DHET)(a), Interleukin-6 (IL-6)(b) and Interleukin-8 (IL-8)(c), over time on Intensive Care for patients with pneumonia (dark line), brain injury without infection (pale line) and brain injury who develop ventilator associated pneumonia (VAP) (dashed line). VAP represents the time that VAP was diagnosed which was not always the final time point. VAP is compared to the day 6 sample in those without infection as this was the timing of diagnosis in 50% of cases of VAP. The table gives the number of samples at each time point. Data are displayed as median with interquartile range.

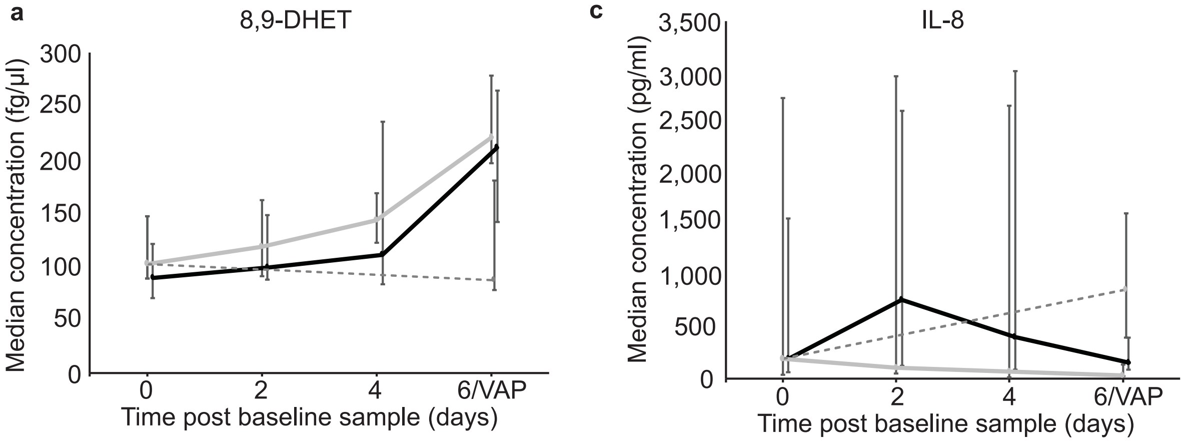

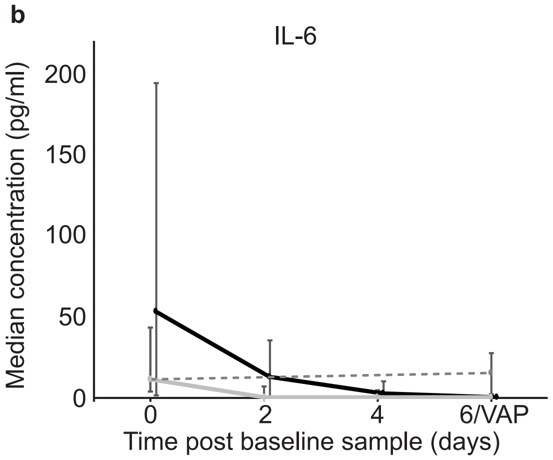

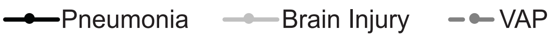

	Day 0	Day 2	Day 4	Day 6/VAP diagnosis
Pneumonia	17	14	12	8
Brain injury	27	13	7	6
VAP		-	-	8
